# Research progress on immunometabolism and gut microbiota in cryptococcal meningitis: mechanisms and therapeutic implications

**DOI:** 10.3389/fnins.2025.1622349

**Published:** 2025-07-16

**Authors:** Sha Wen, Mu Liu, Chengyu Pan, Linhai Zhang, Rong Yan, Zucai Xu

**Affiliations:** ^1^Department of Neurology, Affiliated Hospital of Zunyi Medical University, Zunyi, China; ^2^Zhejiang Provincial People's Hospital Bijie Hospital, Bijie, China; ^3^Collaborative Innovation Center of Tissue Damage Repair and Regeneration Medicine, Zunyi, China; ^4^Key Laboratory of Brain Function and Brain Disease Prevention and Treatment of Guizhou Province, Zunyi, China

**Keywords:** cryptococcal meningitis (CM), immunity, metabolism, gut microbiota, gut-brain axis

## Abstract

Cryptococcal meningitis (CM) is a fatal central nervous system infection caused by *Cryptococcus neoformans* breaching the blood–brain barrier (BBB), carrying a mortality rate approaching 100% in untreated individuals, while even survivors following treatment often experience neurological complications including optic nerve atrophy, memory impairment, hydrocephalus, and motor dysfunction. Current research has yet to fully elucidate the complex pathological mechanisms of CM, particularly leaving a significant gap in the systemic analysis within the dynamic interaction network of immunity, metabolism, and the gut microbiota. This article systematically integrates the interplay of immune responses, metabolic reprogramming, and the gut microbiome to reveal the pathogenesis of CM across multiple dimensions: in immune regulation, the phagocytic-inflammatory equilibrium in macrophages and CD4 + T cells defends against pathogen invasion, but hyperactivated immune responses may damage the BBB and exacerbate neural injury; metabolically, host iron overload induces ferroptosis, disrupting the BBB via lipid peroxidation, while inositol metabolism provides substrates for cryptococcal capsular synthesis, enhancing its virulence and promoting CNS invasion; the gut microbiota, meanwhile, modulates immune homeostasis via the “gut-brain axis,” with its metabolites (e.g., short-chain fatty acids) enhancing BBB integrity and suppressing neuroinflammation through immunomodulation. We propose a combined therapeutic strategy of “immunomodulators + metabolic inhibitors + microbiota intervention,” moving beyond traditional single-factor research paradigms to establish a multi-omics integrated framework for the precise treatment of CM—spanning molecular mechanisms to clinical translation—and propelling the field of neuroinfectious diseases towards a host-pathogen-microenvironment systemic regulation paradigm.

## 1 Introduction

**Figure 1:**
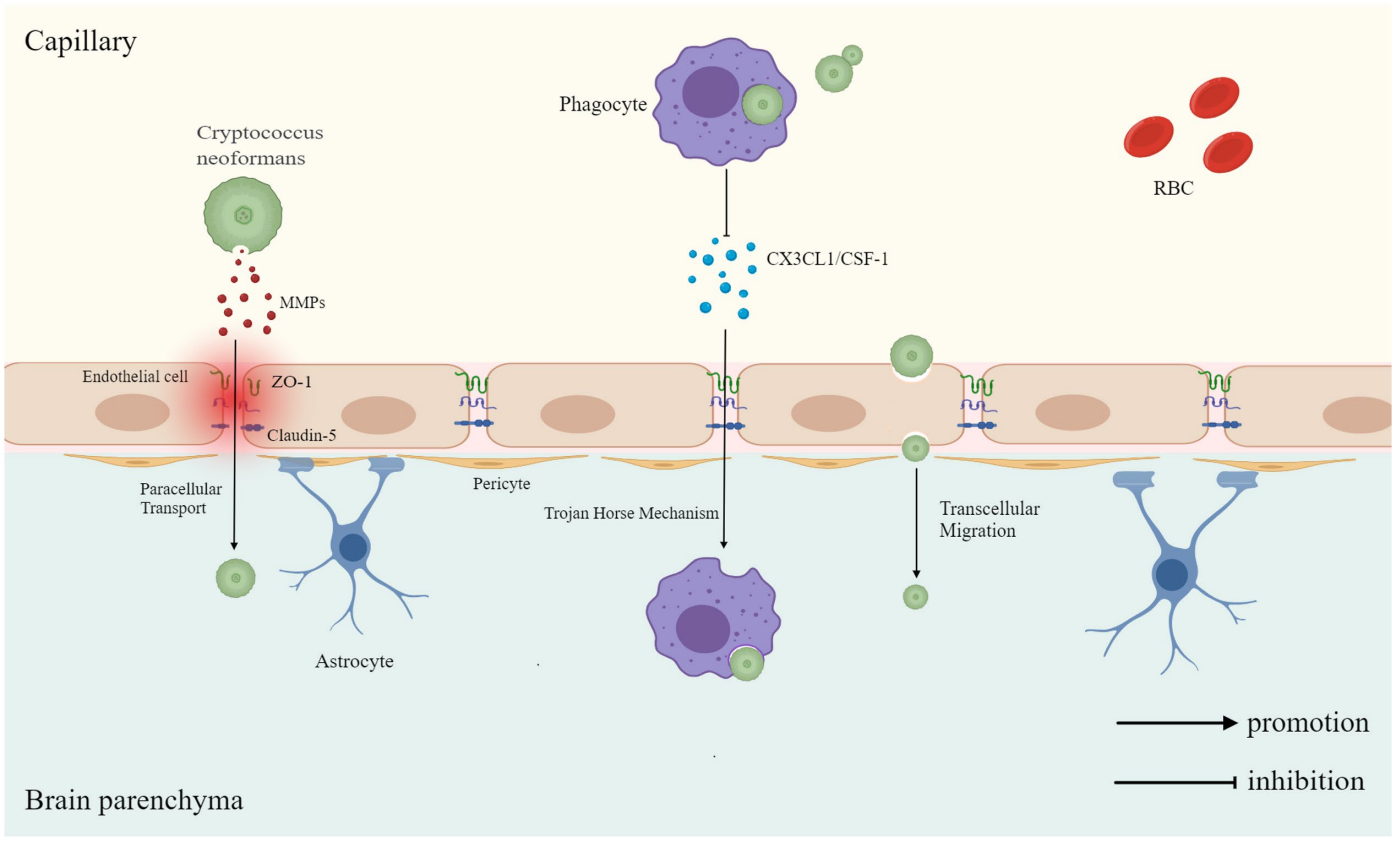
This figure illustrates three mechanisms by which *Cryptococcus neoformans* crosses the blood–brain barrier (BBB): through paracellular transport, where *C. neoformans* secretes matrix metalloproteinases (MMPs) to degrade tight junction proteins zonula occludens-1 (ZO-1) and Claudin-5, disrupting endothelial cell junctions to enter the brain parenchyma; in the Trojan horse mechanism, *C. neoformans* is phagocytosed by phagocytes, and during migration, the phagocyte’s response to endothelial chemokines such as fractalkine (CX3CL1) and colony stimulating factor 1 (CSF-1) is suppressed, slowing cellular migration and facilitating fungal transit across the BBB; transcellular migration involves *C. neoformans* directly traversing the endothelial cell to enter the brain.

*Cryptococcus* is a basidiomycete genus comprising over 30 known species. In nature, two primary species, *C. neoformans* and *Cryptococcus gattii*, are pathogenic to humans (Elsegeiny et al., 2018; Spadari et al., 2018). *Cryptococcus gattii* primarily infects immunocompetent hosts (Chen et al., 2014), while *C. neoformans* mainly affects individuals with HIV infection or other immunocompromised conditions (Baddley et al., 2021; Chen et al., 2023). As a globally distributed opportunistic fungal pathogen, *C. neoformans* ranks high on the World Health Organization’s Fungal Priority Pathogens List. It primarily enters the human body through the respiratory tract, initially colonizing the lungs (Zhao et al., 2023; Saidykhan et al., 2022), and then disseminating from the lungs in immunocompromised hosts (Alanio, 2020), leading to disseminated disease. *C. neoformans* is the leading cause of fatal cryptococcal meningoencephalitis (Aaron et al., 2020; Shourian and Qureshi, 2019; Vidal et al., 2016). CM occurs when *C. neoformans* breaches the BBB and affects the CNS (Zaragoza, 2019), achieved through transcellular traversal, paracellular pathways, and the “Trojan horse” mechanism (Zhou et al., 2024) (Figure 1). CM has been reported in organ transplant recipients, patients with autoimmune diseases on long-term immunosuppressive therapy, individuals with unexplained immunodeficiency, and even healthy individuals (Pappas, 2013; La Hoz and Pappas, 2013; Beardsley et al., 2019). CM is the most common cause of adult meningitis globally, with approximately 250,000 cases annually, resulting in 181,000 deaths. Without treatment, the mortality rate reaches 100% (Zhou et al., 2024). *C. neoformans* most commonly affects HIV-infected individuals, causing HIV-associated CM (HIV-CM) (Tugume et al., 2023), which is the leading cause of death among HIV patients and the second leading cause of HIV-related deaths globally (Jarvis et al., 2014; Jarvis et al., 2022). The WHO reports that *C. neoformans* accounts for about 19% of HIV-related deaths, with CM contributing to 10–15% of global AIDS-related mortality (Francis et al., 2024). In HIV-negative individuals, although the incidence of CM is relatively low, the mortality rate is equally high as in HIV-CM (Williamson et al., 2017). CM poses a severe global mortality risk, particularly in resource-limited developing countries. Despite significant research efforts, no effective preventive vaccine has been developed (Ma et al., 2023). Mortality in patients receiving existing combination antifungal therapy remains as high as 24% at 10 weeks (Iyer et al., 2021). Even the latest research showing that a single high-dose regimen of liposomal amphotericin B combined with flucytosine and fluconazole, while superior to the current standard, can only reduce mortality to below 30% (Jarvis et al., 2022). Studies also indicate that antiretroviral therapy (ART) can restore protective immunity by reducing viral load and reconstituting CD4 + T cells, expanding treatment accessibility; however, associated mortality remains persistently high (Eschke et al., 2015). Furthermore, survivors often experience persistent neurological sequelae such as memory loss, visual deficits, hearing and speech impairments, and motor deficits (Neal et al., 2017), highlighting the need to focus on both acute-phase treatment and long-term neurological function preservation.

In recent years, the research perspective on CM is undergoing a significant paradigm shift. As a lethal central nervous system infection, its treatment faces challenges such as antifungal drug resistance, limited BBB penetrability, and immune reconstitution inflammatory syndrome (IRIS) (Perfect et al., 2010). The study of its pathological mechanisms requires the integration of prevention, early biomarker identification, and precision diagnosis and treatment strategies. Within the pathological process, the immune system plays a defensive role by regulating blood–brain barrier integrity. The newly discovered “gut-brain axis” mechanism suggests that the gut microbiota can influence CM progression by modulating immune homeostasis. Current research is shifting from a sole focus on pathogen eradication towards analyzing the host–microbe interaction network, with particular emphasis on the heterogeneous mechanisms underlying HIV-CM (characterized by CD4 + T cell depletion) versus CM in immunocompetent hosts (dominated by autoimmune abnormalities). In this review, we focus on analyzing the molecular interaction network of the “immune-metabolism-microbiota axis” and explore multimodal therapeutic strategies combining antifungal treatment with immune modulation and microbiota intervention. This review aims to provide a theoretical basis for targeted treatment strategies addressing key pathological links during CM infection, while also offering novel interventions to improve neurological functional outcomes in survivors.

## 2 Host-*Cryptococcus neoformans* immune battle: synergistic defense and immune evasion mechanisms of innate and adaptive immunity

### 2.1 Innate immunity: frontline defense mechanisms

#### 2.1.1 Intracellular evasion mechanisms of cryptococcus

The pathological progression of CM is closely related to the efficacy of the host’s innate immune response, which establishes a critical barrier through multi-layered defense mechanisms in the early stages of infection. *C. neoformans* primarily colonizes the lungs via the respiratory tract and can reactivate and disseminate to the CNS in immunocompromised states (e.g., HIV infection) (Zhou et al., 2024; Yu et al., 2021). During this process, the host initiates a precisely regulated innate immune network involving multiple immune cells and molecular pathways (Table 1). The innate immune system, as the first line of defense against *C. neoformans* infection, employs various immune cells, including macrophages, monocytes, microglia, neutrophils, dendritic cells (DCs), and astrocytes (Campuzano and Wormley, 2018; Mukaremera and Nielsen, 2017). Monocytes/macrophages, neutrophils, and DCs recognize *C. neoformans* pathogen-associated molecular patterns (PAMPs) through pattern recognition receptors (PRRs) such as Toll-like receptor 2 (TLR2) and C-type lectin receptors (CLRs), initiating phagocytosis and inflammatory cascades (Fu and Drummond, 2020) (Table 1).

**Table 1:** Immune cell functions and intercellular interaction mechanisms in cryptococcal meningitis.

Cell type	Functions	Mechanisms	Main impact on BBB	References
Microglial Cells	M1: Microbicidal Activity/M2: Immunomodulatory Function	Release TNF-α and IL-10 in concert with astrocytes	Excessive activation induces increased BBB permeability	Chen Y. et al. (2022), Ginhoux et al. (2010), Vainchtein and Molofsky (2020), Kwon and Koh (2020), Sun et al. (2023)
Astrocytes	Maintenance of BBB integrity and secretion of NO	Promotion of BBB reconstitution	Homeostatic maintenance vs. exuberant immunopathology	Woo and Martinez (2021), Wang et al. (2022), Vainchtein and Molofsky (2020); Zha et al. (2024)
Dendritic Cells	Induction of naive T cell differentiation	Antigen presentation to T cells initiates and orchestrates the adaptive immune response	Augmentation of pathogen clearance	Campuzano and Wormley (2018), Fu and Drummond (2020), Nelson et al. (2021), Guasconi et al. (2022)
Macrophages/ Monocytes	Phagocytic activity in combination with IFN-γ-dependent fungicidal mechanisms.	Phagocytosis and antigen presentation activate both adaptive immunity and trained immunity.	The “Trojan horse” mechanism facilitates fungal traversal across the blood–brain barrier (BBB) via intracellular carriage	Campuzano and Wormley (2018), Fu and Drummond (2020), Chen Y. et al. (2022), Burgess et al. (2022), Das Gupta et al. (2016), Yang et al. (2022), Guilliams et al. (2018)
T Cells	Pathogen containment coupled with orchestrated immune modulation	CD8^+^ T cells release inflammatory mediators; microglia modulate T cell activity.	Neuroprotective homeostasis versus hyperactivation-induced BBB compromise	Woo and Martinez (2021), Mohamed et al. (2022), Rizzo et al. (2020), Dong (2021), Sun et al. (2023)
B Cells	Elicitation of anti-capsular antibodies	Antigen presentation drives effector T cell differentiation	Indirect orchestration of neuroimmune homeostasis	Chen et al. (2023), Okurut et al. (2020), Davis and Lionakis (2018), Bao and Cao (2014)

*Cryptococcus neoformans* employs sophisticated intracellular survival strategies and immune evasion mechanisms after being phagocytosed by monocytes or macrophages. Its thick polysaccharide capsule serves as the first protective barrier, blocking phagosome-lysosome fusion to prevent exposure to acidic hydrolases. Simultaneously, melanin produced by the fungus exerts potent antioxidant effects, effectively neutralizing microbicidal substances such as reactive oxygen species and nitric oxide released by macrophages, thereby maintaining its intracellular integrity (Feldmesser et al., 2001; de Castro et al., 2023; Johnston and May, 2013). More critically, *Cryptococcus* secretes specific effector molecules to modulate host cell polarization, promoting macrophage transformation into non-protective M2 phenotypes. This suppresses their microbicidal functions while enhancing tissue repair and immunosuppressive capacity (Davis et al., 2013; Subramani et al., 2020) This ‘Trojan horse’ strategy allows *Cryptococcus* not only to survive and proliferate safely within macrophages but also to exploit the migratory properties and tissue-penetrating ability of host cells. Carried by infected cells, it traverses physiological barriers. Through these coordinated molecular mechanisms, *Cryptococcus* invasively breaches the anatomical defense of the BBB, reaches the brain parenchyma to establish colonization, and triggers neuroinflammation mediated by neuronal degeneration and glial cell activation.

#### 2.1.2 Regulation of blood–brain barrier function by glial cells and their dual role in cryptococcal meningitis

**Figure 2:**
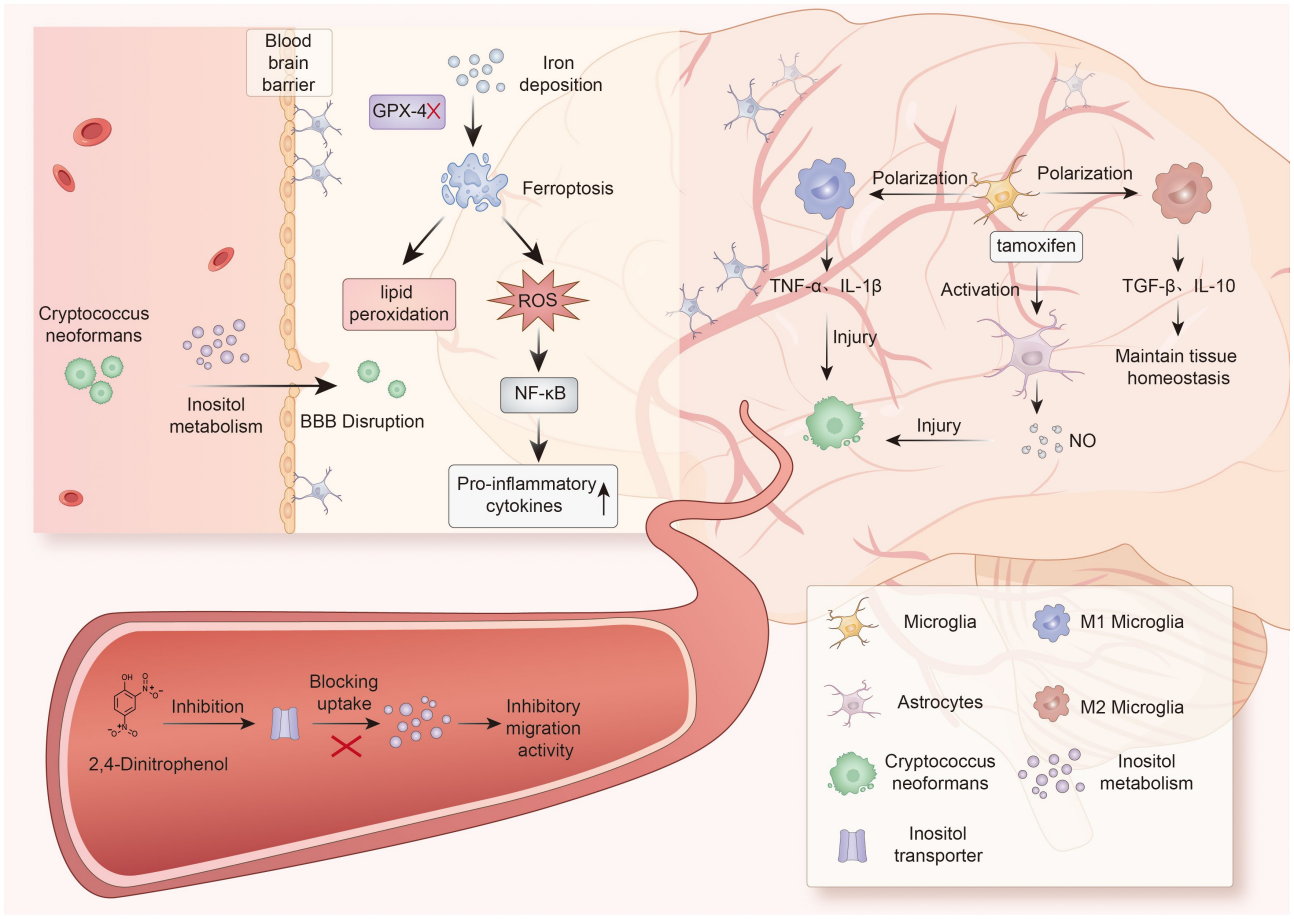
This figure illustrates the molecular mechanisms of inositol metabolism, ferroptosis, and neuroinflammation in *Cryptococcus neoformans* infection: *C. neoformans* enhances its virulence through inositol metabolism, while 2,4-Dinitrophenol, a specific inhibitor of the inositol transporter, blocks fungal inositol uptake and suppresses its migratory activity. Iron deposition induces ferroptosis, characterized by inactivation of glutathione peroxidase 4 (GPX4), accumulation of lipid peroxidation, and reactive oxygen species (ROS) production, which ultimately promotes pro-inflammatory cytokine secretion by activating the NF-κB signaling pathway, thereby disrupting blood–brain barrier (BBB) integrity. Microglial polarization occurs: M1-type microglia release pro-inflammatory factors such as TNF-*α* and IL-1β, causing tissue damage, while M2-type microglia secrete TGF-β and IL-10 to maintain tissue homeostasis. Tamoxifen activates astrocytes to produce nitric oxide, exerting anti-cryptococcal effects.

Astrocytes and microglia play supportive and regulatory roles in maintaining the structure and function of the BBB: Astrocytes secrete vascular endothelial growth factor and Wnt growth factors to help preserve BBB integrity; Microglia, as resident immune cells of the central nervous system, can directly phagocytose or kill pathogenic microorganisms that enter the brain parenchyma (Haruwaka et al., 2019; Guérit et al., 2021; Proia et al., 2008). Specifically, during CM pathogenesis, microglia dynamically switch between M1/M2 polarization states: M1 microglia directly kill *C. neoformans* by secreting pro-inflammatory cytokines such as TNF-*α* and IL-1*β*, while M2 microglia maintain tissue homeostasis through secretion of IL-10 and TGF-β (Orihuela et al., 2016; Nielson and Davis, 2023). Calcium (Ca^2+^) signaling between astrocytes constitutes a key mechanism regulating BBB permeability. Through gap junctions forming functional networks, Ca^2+^ fluctuations rapidly propagate between cells, coordinating the expression and distribution of BBB tight junction proteins (De Bock et al., 2016; Paemeleire, 2002). During *C. neoformans* infection, astrocytes are activated and produce nitric oxide, which not only exerts direct anti-cryptococcal effects but also modulates vasoconstriction and BBB permeability (Lee et al., 1994). However, excessive activation of the inflammatory response often further damages the BBB, leading to increased infiltration of immune cells and release of neurotoxic inflammatory mediators. This causes brain injury and exacerbates the pathological progression of CM (Woo and Martinez, 2021; Huang et al., 2012). Activated immune cells and the excessive secretion of cytokines (e.g., IFN-*γ*, TNF-α) may also damage BBB tight junction proteins, increasing its permeability (Mukaremera and Nielsen, 2017; Al-Huthaifi et al., 2024). Although immune cells can resist *C. neoformans* infection, excessive inflammatory responses may disrupt BBB function. Therefore, in CM treatment and prognosis, besides considering antifungal drugs to eliminate the pathogen, it is essential to regulate immune cell function and inflammatory factor balance to prevent BBB damage and neurological dysfunction (Figure 2).

### 2.2 Adaptive immune defense mechanisms and early intervention

The host’s anti-*C. neoformans* immune defense system forms a synergistic network through PRR-mediated innate immunity and antigen-specific adaptive immunity. DCs act as immune hubs, not only clearing pathogens through phagolysosome maturation but also activating T lymphocyte clonal expansion via MHC-II-dependent antigen presentation, completing the spatiotemporal transition of immune responses (Coelho, 2024; Pacifici et al., 2023; Pishesha et al., 2022). In adaptive immunity, CD4 + T lymphocytes play a central regulatory role by polarizing into Th1-type immune phenotypes, establishing antifungal immune surveillance and pathogen clearance. Adaptive immunity achieves three functions through the synergistic interaction of antigen-presenting cells (APCs) and lymphocytes: antigen presentation triggering specific activation of T and B lymphocytes, CD4 + and CD8 + T cell-mediated cellular immune responses promoting early fungal clearance, memory cell formation providing long-term protection, and dynamic balance between defense responses and self-tolerance through immune regulatory networks (Bonilla and Oettgen, 2010). Triggering adaptive immunity requires the uptake, processing, and antigen presentation by immune cells such as macrophages, DCs, and monocytes (Mukaremera and Nielsen, 2017). CNS immune responses are regulated by dual mechanisms: on one hand, resident immune cells (microglia, astrocytes) and peripheral infiltrating cells (monocytes, T/B lymphocytes) form a dynamic regulatory network, collectively regulating BBB permeability; on the other hand, the BBB strictly maintains CNS immune homeostasis by limiting cell migration and regulating molecular transport (Fu and Drummond, 2020; Mohamed et al., 2022; Hazra et al., 2019). APCs (e.g., DCs and macrophages) present processed *C. neoformans* antigens to T cells via MHC-II molecules (Mohamed et al., 2022) Studies show that CD4 + T cell-derived IFN-*γ* significantly enhances macrophage bactericidal function by activating the STAT1 signaling pathway (Leopold Wager et al., 2018). Batf3-dependent classical type 1 DCs (cDC1) play a central role in generating IFN-γ + CD4 + T cells, increasing tissue-resident memory T cell (TRM) numbers by 2–3 times, thereby enhancing antifungal immune defense (Coelho, 2024). Notably, the host has established a multi-layered immune response regulatory network. IFN-γ, as a core mediator of Th1-type immune responses, plays a key role in breaking pathogen immune evasion. The monocyte–macrophage system and neutrophils rapidly secrete IFN-γ through STAT1-dependent signaling pathways. This transcription factor binds to the IFN-γ receptor, activating downstream JAK–STAT signaling cascades and promoting antimicrobial gene expression. This STAT1-mediated early pro-inflammatory response has dual effects: it enhances immune surveillance through mechanisms such as antigen presentation and oxidative burst, but excessive production of inflammatory mediators like TNF-*α* may increase BBB permeability (Wang et al., 2024).

### 2.3 *Cryptococcus neoformans* immune evasion and host countermeasures

In CM’s immune defense mechanisms, *C. neoformans* has evolved complex immune evasion strategies to counteract host innate immunity. *C. neoformans* virulence factors primarily include the polysaccharide capsule, melanin, cell wall integrity, thermotolerance, and secreted enzymes (Iyer et al., 2021). Among these, the polysaccharide capsule and melanin are the two most critical virulence factors, aiding *C. neoformans* survival and growth within the human host (Baker et al., 2024). The polysaccharide capsule modulates host immune responses and enhances pathogenicity, playing a key role in infection. The capsule’s structure, size, and density dynamically change, forming an effective immune evasion mechanism that helps the pathogen evade host immune recognition and clearance (Zaragoza, 2019; Dang et al., 2022; O'Meara and Alspaugh, 2012). Melanin acts as an antioxidant and reactive oxygen species (ROS) scavenger, providing fungal protection against oxidative stress and host defense mechanisms (Cordero et al., 2020; Lee et al., 2019). *C. neoformans* employs structural defense factors (capsule polysaccharides and melanin) to construct physical barriers and resist oxidative stress, counteracting alveolar macrophage phagocytosis. This immune evasion strategy is a key pathogenic mechanism for lung colonization (Lee et al., 2020).

*Cryptococcus neoformans* can increase phagolysosome membrane permeability through phospholipase B1 activity, disrupting lysosomal pH homeostasis and significantly impairing phagocytic bactericidal function (Campuzano and Wormley, 2018; Wang et al., 2024). This unique “immune sanctuary” effect not only promotes pathogen survival but also evades immune surveillance by interfering with lysosome fusion and regulating autophagy pathways, allowing *C. neoformans* to survive and disseminate within the host, creating a microenvironment for latent infection (Mohamed et al., 2022; Wang et al., 2022).

### 2.4 Clinical significance of immune biomarkers in CM

As research progresses, specific immune biomarkers and their dynamic changes play an important role in assessing CM risk and guiding early intervention (Rajasingham et al., 2017). Among various immune biomarkers, CD4 + T cell count is the most classic and practical predictive indicator. When CD4 + T cell counts drop below 100/μL, the risk of CM significantly increases. Therefore, timely monitoring of this indicator and implementing antifungal prophylaxis have become consensus in global clinical guidelines (Pérez-Jacoiste Asín et al., 2021). The combined application of cryptococcal antigen (CrAg) detection and immune biomarkers holds significant clinical value. Although CrAg positivity does not necessarily progress to meningitis, combining it with CD4 + T cell counts and cytokine levels can more accurately identify high-risk populations (Li et al., 2020; Borges et al., 2019; Hailu et al., 2019; Rajasingham et al., 2022). Beyond total counts, functional differences among CD4 + T cell subsets are also noteworthy. Th1, Th2, and Th17 subsets play distinct roles in antifungal immunity and inflammation regulation. Specifically, Th1-type cytokines (e.g., IFN-*γ*) activate macrophages to promote *C. neoformans* clearance, while Th2-dominated immune responses may indicate excessive inflammation risks (Pawlak et al., 2020; Firacative et al., 2018; Shourian et al., 2017; Balasko and Keynan, 2019; Jarvis et al., 2015). Recent studies suggest that CD8 + T cell interactions with the monocyte–macrophage system are also crucial for infection defense, with activated macrophage phenotypic changes reflecting the host’s innate immune status (Subramani et al., 2020; Meya et al., 2015). In the cytokine network, IFN-*γ*, IL-6, and TNF-*α* not only participate in infection control but also correlate with disease severity. Detecting cytokine levels in blood or cerebrospinal fluid (CSF) can predict disease progression trends early (Okafor et al., 2024; Mora et al., 2015; Xu L. et al., 2021). As research on immune biomarkers deepens, their application in CM early warning and prevention will become more precise, offering new strategies to reduce disease-related mortality.

### 2.5 Immunological perspectives on CM prevention and treatment

#### 2.5.1 Immunomodulatory therapy with monoclonal antibodies and cytokines

CM is a severe CNS infection, and immunomodulatory therapy offers new insights and strategies. In recent years, significant progress has been made in exploring CM immunotherapies, including monoclonal antibodies, cytokines, immunomodulators, and vaccines. Monoclonal antibodies and cytokines have shown unique potential in CM treatment. Monoclonal antibodies targeting specific antigens can enhance the immune system’s ability to recognize and clear *C. neoformans*. For example, monoclonal antibodies against *C. neoformans* capsule polysaccharides can neutralize its pathogenicity and promote phagocyte uptake (Larsen et al., 2005). Cytokines can modulate immune cell function, restoring immune balance. IFN-γ, for instance, enhances macrophage anti-cryptococcal activity, improves fungal clearance in CSF, promotes protective immune responses, and improves survival in HIV-CM patients (Zhang et al., 2022; Tao et al., 2022). Some immunomodulators can also enhance immune responses while reducing inflammatory tissue damage. A prospective study on the immunomodulator lenalidomide showed it significantly reduced serum TNF-α and IL-6 levels, improving intracranial inflammation (Wan et al., 2023). The efficacy of immunomodulatory therapy is closely related to the patient’s immune status, with patients having higher baseline IFN-γ levels often having better prognoses (Jarvis et al., 2013). Combining this therapy with traditional antifungal drugs may improve outcomes.

#### 2.5.2 Development strategies and immune mechanisms of cryptococcal vaccines

Vaccine development for CM is a strategic direction in immunotherapy, aiming to synergistically activate host innate and adaptive immune responses to establish long-term defense against *C. neoformans*. Studies have shown that candidate vaccines based on key antigen components such as glucuronoxylomannan and sterylglucoside exhibit dual immune activation properties in animal models: they activate innate immune responses through DC PRRs (e.g., TLR4/MyD88 signaling) and induce Th1-type cellular immunity and memory B cell differentiation through HLA-II molecule presentation, achieving both infection prevention and recurrence control (Colombo et al., 2019). Further research found that a heat-killed *prm1* deletion strain vaccine activates alveolar macrophage phagocytosis and promotes Th1/Th17 cell polarization, significantly enhancing CNS immune surveillance, reducing brain fungal load, and extending protection (Li C. et al., 2023). This vaccine design not only avoids the proliferation risks of live vaccines but also demonstrates excellent biosafety, offering an innovative strategy for precise CM immunomodulation with long-term protection and clinical safety.

#### 2.5.3 Astrocyte-based therapeutic targets for neuroprotection

Significant progress has been made in CM immunotherapy target research, such as neuroprotective strategies based on astrocyte function, which have become an important research direction in CM prevention and treatment. These strategies show potential therapeutic value by regulating BBB integrity and inhibiting neuroinflammation (Woo and Martinez, 2021). Studies have explored tamoxifen’s ability to induce astrocyte-specific activation, exerting antifungal activity. Tamoxifen has also been shown to induce astrocyte-specific activation, exerting antifungal effects (Wang et al., 2023).

## 3 Metabolic and immune interactions in *Cryptococcus neoformans* infection pathogenesis

### 3.1 Inositol metabolism-mediated capsule virulence synergistically promotes CM

Inositol metabolism is crucial for fungal virulence, primarily due to its impact on *C. neoformans* polysaccharide capsule formation (Wang et al., 2021). It interferes with immune recognition, protects newly formed *C. neoformans* from macrophage phagocytosis, impairs monocyte function, and hinders effective immune cell targeting (Fu and Drummond, 2020; Jang et al., 2022). However, inositol can also induce capsule enlargement and serves as the sole carbon source for *C. neoformans*, playing a key role in fungal development, mating, and virulence. Inositol catabolism releases inositol, providing energy for *C. neoformans* and necessary substrates for polysaccharide capsule formation, thereby enhancing fungal virulence (Wang et al., 2021; Liao et al., 2018). Elevated intracerebral inositol levels stimulate blood–brain barrier endothelial cells to increase hyaluronic acid (HA) production (Liu et al., 2013), HA acts as a ligand binding to the CD44 receptor on endothelial cell surfaces (Jong et al., 2008; Jong et al., 2012), promoting *C. neoformans* adhesion to the vascular side of the BBB (Huang et al., 2011). This enhances the interaction between the fungus and brain microvascular endothelial cells (Chen Y. et al., 2022). Furthermore, HA contributes to BBB disruption by compromising tight junctions between endothelial cells. Collectively, these mechanisms facilitate trans-BBB transport of *Cryptococcus*, ultimately leading to the development of CM (Figure 2).

### 3.2 Iron metabolism-immune interactions drive CM pathogenesis

Iron metabolism-immune interactions play a bidirectional regulatory role in CM pathogenesis, involving dynamic competition between host and pathogen for iron and the resulting expression of *C. neoformans* virulence and host immune defense efficacy. As a key nutrient required by both host immune cells and pathogens, iron uptake determines the course of infection (Do et al., 2020). The host employs a hepcidin-mediated iron restriction strategy to inhibit *C. neoformans* capsule synthesis by suppressing Lac1 activity, weakening immune evasion. In low-iron microenvironments, the pathogen activates high-affinity iron uptake systems, upregulating capsule polysaccharide synthesis genes to evade host PRR immune recognition, forming a dual metabolic-immune defense barrier (Jung et al., 2010; Li et al., 2024). Notably, this competitive imbalance in iron homeostasis can trigger cascading pathological effects: on one hand, host intracellular iron overload triggers ferroptosis through ACSL4-dependent pathways—a form of iron-dependent programmed cell death characterized by glutathione peroxidase 4 inactivation and abnormal lipid peroxidation accumulation, directly disrupting BBB endothelial tight junctions (Dixon and Olzmann, 2024; Xie et al., 2016). On the other hand, iron metabolism dysregulation, coupled with lipid and amino acid metabolic reprogramming, exacerbates oxidative stress damage associated with ferroptosis, forming a vicious cycle of “iron accumulation-lipid peroxidation-BBB permeability increase” (Xu X. et al., 2021; Chen et al., 2021; Xu et al., 2024). The excessive ROS and lipid peroxidation products generated through this process exacerbate CNS infection through dual mechanisms: first, by disrupting BBB tight junctions through oxidative stress, increasing permeability, and promoting *C. neoformans* CNS invasion and inflammatory factor diffusion; second, by modulating immune cell functional states, ROS and lipid peroxidation products activate pro-inflammatory signals (e.g., NF-κB), significantly exacerbating CM-related neuroinflammation, forming a “oxidative damage-neuroinflammation” positive feedback loop (Chen X. et al., 2022) (Figure 2). Additionally, microglial immune surveillance function is closely related to iron metabolism balance. Clinical studies show that abnormal increases in CSF ferritin levels impair microglial phagocytic function and cytokine secretion. This iron metabolism-immune regulatory axis dysfunction may create an immune-privileged microenvironment for CNS infections, involving multiple molecular mechanisms such as transferrin receptor signaling pathway abnormalities and iron regulatory protein (IRP) activity changes (Xu X. et al., 2021; Rochette et al., 2022; Latunde-Dada, 2017).

### 3.3 Oxidative stress metabolism and immune synergistic regulation of *Cryptococcus neoformans* pathogenicity

During host infection, *C. neoformans*-immune system interactions are regulated by oxidative stress metabolism. *C. neoformans* dynamically regulates energy metabolism to adapt to the host microenvironment. Under hypoxic conditions, it relies on transcription factors Pas2 and Rds2-mediated metabolic reprogramming, inhibiting mitochondrial oxidative phosphorylation and activating glycolysis to reduce ROS generation and maintain CNS invasion capability (Zhao and Lin, 2021). Host immune cells (e.g., neutrophils, macrophages) produce large amounts of ROS through respiratory bursts to clear pathogens. However, excessive ROS not only damages host cells, causing oxidative stress, but also chronically activates immune cells, forming a vicious cycle of ROS overproduction. To counteract oxidative damage, *C. neoformans* employs multi-layered antioxidant mechanisms, including upregulating superoxide dismutase, glutathione systems, and melanin synthesis to neutralize ROS, while utilizing inositol metabolism to maintain membrane integrity and evade immune recognition (Spadari et al., 2018) (Figure 2).

### 3.4 Metabolic-immune synergistic regulatory network strategies for CM prevention and treatment

In *C. neoformans*, inositol pyrophosphates (PP-IPs) are key metabolites for adapting to the host environment. Inhibiting their biosynthesis can weaken fungal virulence, making PP-IP metabolic pathways potential targets for antifungal drug development (Kang, 2018). The inositol pyrophosphate pathway, as a key metabolic signaling hub in *C. neoformans*, regulates fungal cell cycle, virulence factor secretion, environmental stress adaptation, and host immune evasion mechanisms, participating in the dynamic balance of its pathogenic processes (Gogianu et al., 2024). Among these, inositol polyphosphate kinase Kcs1 negatively regulates inositol uptake and metabolism in *C. neoformans*. Knocking out this gene reduces *C. neoformans* CNS invasion ability, suggesting its potential as a drug target (Liao et al., 2018) (Table 2). *C. neoformans* utilizes multiple inositol transporters and host inositol-dependent transport systems to synergistically promote BBB penetration. Dinitrophenol (DNP), a specific inhibitor of inositol transporters, blocks *C. neoformans* inositol uptake, inhibiting migration activity and significantly reducing pathogenic virulence (Liu et al., 2013; Porollo et al., 2014) (Table 2). In oxidative stress-immune regulation intervention strategies, J-domain proteins Mrj1 and Mar1 regulate mitochondrial respiratory chains and ROS homeostasis, directly impacting fungal virulence and antifungal tolerance, making them potential therapeutic targets (Calderone et al., 2015; Horianopoulos et al., 2020; Telzrow et al., 2023) (Table 2). Basic leucine zipper protein Gsb1 and heat shock factor HSF have been shown to regulate ROS scavenging genes and mitochondrial homeostasis, weakening *C. neoformans* virulence (Cheon et al., 2017; Gao et al., 2022).

**Table 2:** Key agents for the triad therapy in cryptococcal meningitis and their experimental/clinical evidence status.

Therapeutic category	Agent/Intervention	Mechanism of action	Experimental/Clinical evidence level	Key findings	References
Immunomodulators	IFN-γ	activates STAT1 signaling pathway	Phase III clinical trial	Increased CSF cryptococcal clearance rate	Jarvis et al. (2012), Pappas et al. (2004)
	Monoclonal Antibody 18B7	Neutralization of the polysaccharide capsule via specific antibodies	Phase I clinical trial	Reduction in serum cryptococcal antigen titers	Larsen et al. (2005)
	Lenalidomide	Reduces TNF-α and IL-6 levels	Prospective clinical study	Mitigation of intracerebral inflammation	Wan et al. (2023), Liu et al. (2022)
Metabolic Inhibitors	DNP	Inhibition of myo-inositol acquisition	Preclinical study	Inhibition of BBB penetration	Liu et al. (2013), Porollo et al. (2014)
	Inositol polyphosphate kinase kcs1 inhibitor	Targeting myo-inositol metabolic flux to suppress virulence	Basic research	Potential drug targets	Liao et al. (2018)
	Hepcidin Mimetic PR-73	Mimics hepcidin to restrict iron availability, inhibits iron-dependent growth/virulence	Animal model study	Reduction in infection-attributable mortality	Arekar et al. (2024)
Microbiota interventions	Mrj1/Mar1 (J-domain protein) Inhibition	Respiratory chain dysfunction	Basic research	Reduces fungal virulence	Calderone et al. (2015), Horianopoulos et al. (2020), Telzrow et al. (2023)
	Probiotics	Restores gut microbiota; suppresses inflammation	Basic Research	Enhances BBB integrity;	Li H. J. et al. (2023), Loh et al. (2024), Fock and Parnova (2023)
	SCFAs	GPR43/41 receptor agonism	Basic research	Suppression of neuroinflammation	Li H. J. et al. (2023), Loh et al. (2024), Fock and Parnova (2023), Parker et al. (2020), Benakis et al. (2016), Nakajima et al. (2017), Saikachain et al. (2023)

## 4 Integrated mechanisms of the lung-gut-brain immune-metabolic axis in cryptococcal meningitis (CM)

Following *Cryptococcus* infection, the fungus first colonizes and rapidly proliferates within the alveoli, activating alveolar macrophages and dendritic cells. The latter recognize cryptococcal components via pattern recognition receptors (such as Toll-like receptors), prompting the secretion of high levels of pro-inflammatory cytokines including IL-6, TNF-*α*, and IFN-*γ* (Korkmaz and Traber, 2023; Ye et al., 2025). These inflammatory cytokines enter the intestinal tissue and brain via the circulatory system. In the gut, these factors not only disrupt tight junction proteins (e.g., occludin, claudin-1), weakening the intestinal epithelial barrier and increasing permeability, but also drive a decline in beneficial microbiota (such as *Lactobacillus*) and an increase in opportunistic pathogens (such as *Polymorphobacter*), leading to dysbiosis (Barbara et al., 2021; Chelakkot et al., 2018; Kaminsky et al., 2021). Concurrently, via the CCR2-CCL2 signaling axis, inflammatory monocytes are recruited to cross the vascular wall into the brain tissue, further activating microglia and causing neuroinflammation and BBB damage (Dimitrijevic et al., 2006; Dimitrijevic et al., 2007; Geng et al., 2022). This sequence of events establishes the “lung-gut-brain” axis as a highly coordinated immune crosstalk network, creating favorable conditions for the hematogenous dissemination of *Cryptococcus* and its penetration of the BBB into the central nervous system.

**Figure 3:**
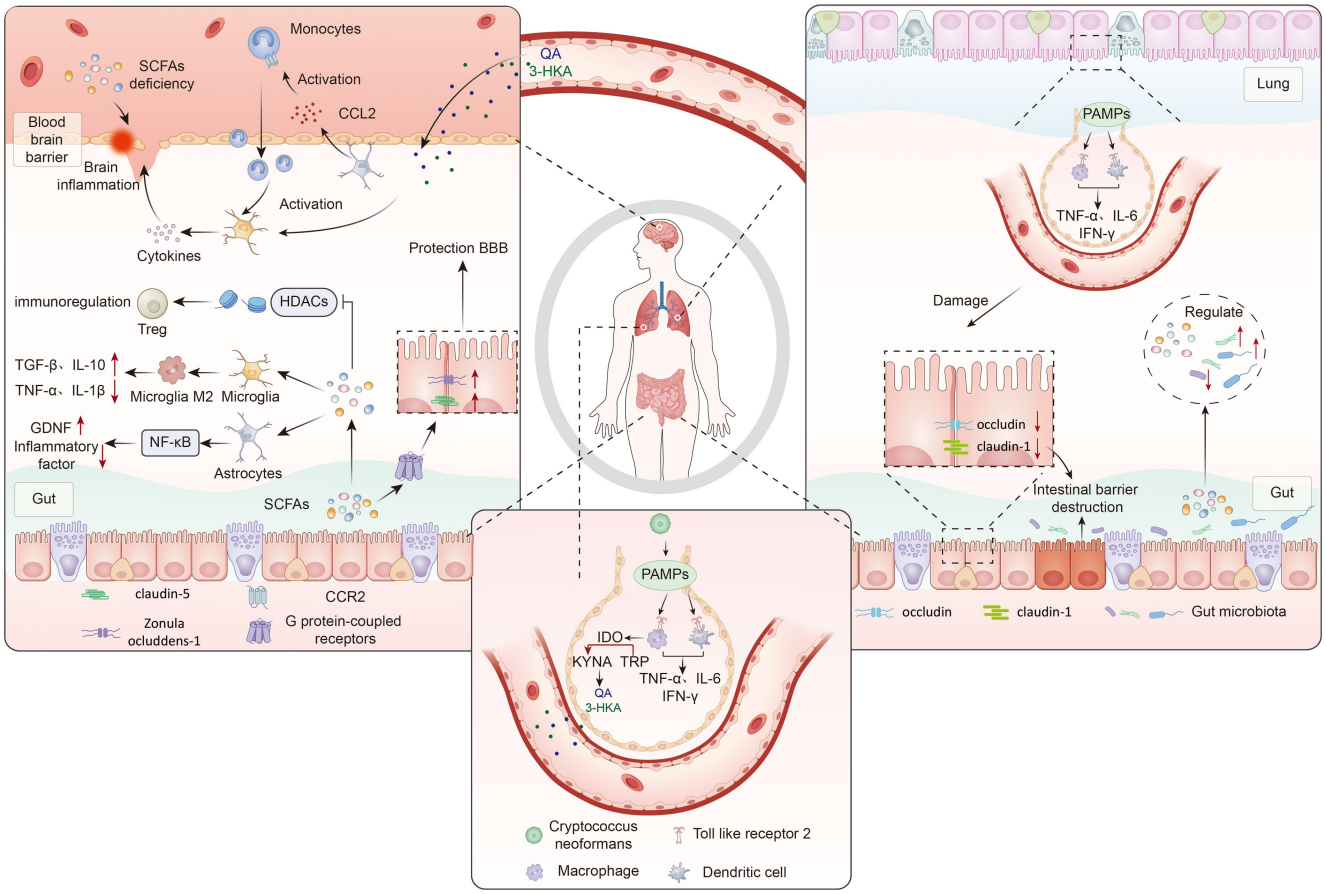
This figure illustrates the brain-gut-lung axis interactions in *Cryptococcus neoformans* infection: After colonizing the lungs, pathogen-associated molecular patterns (PAMPs) of *C. neoformans* activate alveolar macrophages and dendritic cells through Toll-like receptor 2 (TLR2) recognition, inducing secretion of inflammatory cytokines such as TNF-α, IL-6, and IFN-γ. These cytokines disrupt intestinal tight junction proteins (occludin, claudin-1) via the bloodstream, leading to intestinal barrier damage and gut dysbiosis. Altered gut microbiota causes short-chain fatty acid (SCFA) deficiency. SCFAs influence the brain through three pathways: (1) downregulating histone deacetylases (HDACs) to promote regulatory T cell (Treg) differentiation; (2) activating G protein-coupled receptors (GPCRs) to maintain blood–brain barrier (BBB) integrity; (3) driving microglial polarization toward the anti-inflammatory M2 phenotype (suppressing IL-1β and TNF-α release) and promoting astrocytes to upregulate the neuroprotective factor glial cell line-derived neurotrophic factor (GDNF) via the TLR4/NF-κB pathway, establishing an anti-cryptococcal immune microenvironment. Concurrently, inflammatory monocytes are recruited into the brain via CCL2, activating microglia, while neuroactive metabolites from the indoleamine 2,3-dioxygenase (IDO)-kynurenine (KYNA)-quinolinic acid (QA) pathway, such as 3-hydroxykynurenine (3-HKA), impact brain function.

Pulmonary inflammation also activates the IDO-tryptophan-kynurenine pathway, promoting the conversion of tryptophan to kynurenine and its downstream metabolites under IDO catalysis. These metabolites can cross the blood–brain barrier, modulating astrocyte and microglial activity. Simultaneously, by altering intestinal lumen pH and substrate availability, they impact gut microbiota diversity and exacerbate ecological imbalance (Cervenka et al., 2017; Tsuji et al., 2023; Bohn et al., 2024). Changes in the gut microbial community further result in reduced levels of short-chain fatty acids (SCFAs), such as butyrate and propionate. SCFA deficiency severely compromises BBB integrity and neuroimmune homeostasis: On one hand, SCFAs activate G protein-coupled receptors (GPR43/41) to upregulate tight junction protein expression (ZO-1/Claudin-5), while inhibiting histone deacetylase (HDAC) to promote regulatory T cell (Treg) differentiation, thereby preserving BBB integrity through both physical and immune barrier dimensions (Li H. J. et al., 2023); On the other hand, SCFAs (e.g., propionate) drive microglial polarization towards the anti-inflammatory M2 phenotype via the metabolism-immune axis, suppressing the release of pro-inflammatory cytokines like IL-1β and TNF-α. Concurrently, they induce astrocytes to downregulate pro-inflammatory factor secretion and upregulate neuroprotective factors (e.g., GDNF) via the TLR4/NF-κB pathway, creating an immune microenvironment that inhibits *Cryptococcus* proliferation (Li H. J. et al., 2023; Loh et al., 2024; Fock and Parnova, 2023). This immune-metabolic reprogramming not only reduces damage to BBB endothelial cells by inflammatory mediators but also improves BBB permeability by regulating the dynamic balance of matrix metalloproteinases (MMPs) (Parker et al., 2020; Benakis et al., 2016). SCFAs typically enhance the phagocytic and bactericidal capacity of alveolar macrophages and help maintain BBB integrity via the GPR43-AMPK signaling pathway; SCFA deficiency weakens this immune barrier effect and sustains intracerebral inflammation (Nakajima et al., 2017; Saikachain et al., 2023) (Table 2). Furthermore, the pulmonary inflammatory response significantly alters the interaction between bile acid metabolism and the gut microbiota. Abnormal activation of the FXR-FGF19 signaling axis promotes microglial polarization towards the pro-inflammatory M1 phenotype, and the release of neurotoxic factors further damages tissue (Mulak, 2021; Ma et al., 2024) (Figure 3).

Gut microbiota dysbiosis holds significant clinical implications in CM patients. Studies indicate that this dysregulated state can persist long-term, continuously modulating neuroinflammatory responses and neuroplasticity via the gut-brain axis. Notably, persistent gut microbial imbalance in CM survivors is significantly associated with various neurological sequelae. Clinical data demonstrate a positive correlation between the degree of gut dysbiosis and disease severity/prognosis: patients exhibiting reduced microbial diversity and increased proportions of pathogenic bacteria show markedly worse clinical outcomes, with significantly increased mortality risk (Ma et al., 2023; Panackal et al., 2015; Li et al., 2022). Therefore, dynamic monitoring and timely intervention targeting the gut microecology are crucial not only for acute-phase treatment but also for improving long-term neurological functional outcomes in patients.

## 5 Evidence base for immune-metabolic-microbiota interventions

The immune-metabolic-microbiota triad therapy is grounded in the theoretical framework of the multi-faceted pathogenesis of CM. It combines the modulation of host immunity, pathogen metabolism, and the intestinal microbiome, creating a novel therapeutic paradigm for refractory fungal infections. Among the existing evidence, immunomodulators represent the most clinically validated component of the triad intervention. A multicenter randomized controlled trial investigating interferon-gamma (IFN-*γ*) combined with amphotericin B and flucytosine for HIV-associated CM demonstrated an increase in early fungicidal activity (EFA) from −0.49 log CFU/ml/day to −0.64 log CFU/ml/day (*p* = 0.02) (Jarvis et al., 2012) with efficacy observed after only two doses of IFN-γ. Another study involving 75 AIDS-related cases showed that the 2-week CSF culture conversion rates were 36 and 32% in the 100 μg and 200 μg IFN-γ treatment groups, respectively, significantly higher than the 13% rate in the placebo group (Pappas et al., 2004). Mechanistic studies further confirmed that patients with higher baseline IFN-γ levels had superior clinical outcomes (Jarvis et al., 2015; Jarvis et al., 2013). A phase I trial of the monoclonal antibody 18B7 established its maximum tolerated dose at 1.0 mg/kg. While it significantly reduced serum cryptococcal antigen titers by two- to three-fold, its concentration in the CSF was extremely low (Larsen et al., 2005). In contrast, metabolic inhibitors such as inositol transporter inhibitors and the hepcidin mimetic PR-73 have demonstrated antifungal activity in animal models and significantly improved infection outcomes. However, human safety and efficacy clinical data are currently lacking (Arekar et al., 2024) (Table 2).

Regarding microbiota interventions, although fecal microbiota transplantation (FMT) and probiotic interventions are widely used clinically in conditions like ulcerative colitis, with remission rates ranging from 32 to 53% and good tolerability (Costello et al., 2019; Haifer et al., 2022; Paramsothy et al., 2017), direct data in the context of cryptococcal meningitis are absent. FMT has been shown to improve non-motor symptoms in Parkinson’s disease patients and alter brain dopamine transporter binding in metabolic syndrome patients (Cheng et al., 2023; Hartstra et al., 2020), but these findings cannot be directly extrapolated to CM treatment. Supplementation with probiotics aims to maintain intestinal microecological homeostasis, enhance SCFA production, strengthen the gut barrier, and reduce central nervous system inflammation. Key aspects of the triad therapy, including dose optimization, treatment sequencing, and component synergism, still lack systematic clinical validation. Although novel immunomodulators like lenalidomide can significantly reduce cell-associated HIV RNA, improve chronic inflammation, and lead to significant decreases in serum inflammatory markers such as CRP and IL-6 in patients (Wan et al., 2023; Liu et al., 2022) (Table 2), the efficacy and safety of their combined application with metabolic and microbiota interventions remain to be elucidated. Overall, the triad intervention possesses a solid theoretical foundation and partial clinical support. However, high-quality clinical trials are still needed to systematically validate the efficacy and safety of each component and the integrated intervention strategy, aiming to deliver tangible benefits to patients with cryptococcal meningitis.

## 6 Outlook

The occurrence of CM is essentially a cascade reaction of pathogens breaching the host’s multi-dimensional defense system, with the immune system playing a central regulatory role in CM’s pathological progression. Its interactions with metabolic dysregulation and gut microbiota dysbiosis are key mechanisms influencing disease progression. The BBB, as the ultimate defense barrier of the CNS, serves as the synergistic hub of the immune-metabolism-microbiota network: overactivation of immune cells (e.g., microglia), oxidative damage associated with ferroptosis, and microbiota dysbiosis-induced BBB permeability increases collectively constitute the “triple hit” of *C. neoformans* CNS colonization. The neuroimmune unit formed by microglia and astrocytes plays a dual role in BBB dynamic regulation: on one hand, it maintains immune homeostasis through anti-inflammatory factors (e.g., IL-10, TGF-*β*); on the other hand, it initiates inflammatory responses through PRRs. *C. neoformans* breaches the BBB through the “Trojan horse” mechanism, transcytosis, and paracellular pathways, reflecting the failure of host immune surveillance. Pathogen capsule polysaccharides can induce microglial M2 polarization, creating an immunosuppressive microenvironment favorable for *C. neoformans* CNS colonization. Metabolic pathway dysregulation can profoundly reshape immune homeostasis. For example, iron metabolism dysregulation induces BBB dysfunction, inhibits microglial phagocytosis, and exacerbates pro-inflammatory cascades, significantly aggravating CM pathological damage. Meanwhile, *C. neoformans* inositol metabolism enhances pathogenicity by regulating capsule polysaccharide synthesis, evading host immune recognition. The gut microbiota indirectly influences CM progression by modulating the immune system and maintaining BBB integrity, particularly through regulating the functions of microglia and astrocytes. The tripartite interaction system of the microbiota-immune-neuroglia unit restricts the central dissemination of *Cryptococcus* by preserving BBB stability.

Future research must first utilize multi-omics technologies to systematically elucidate the dynamic interaction mechanisms of the “immune-metabolism-microbiota axis” in cryptococcal meningitis, with particular focus on the correlation between blood–brain barrier disruption and neuroinflammation. Building upon this foundation, there is an urgent need to develop temporally precise, stepwise combination therapies—acute phase targeting inositol transport (DNP) synergized with immune modulation (IFN-*γ*) for rapid infection control, recovery phase restoring gut-brain axis homeostasis through probiotics/SCFAs while enhancing central nervous system targeting of drugs (e.g., ferroptosis inhibitors) via nanocarriers. Concurrently, to reverse neurological damage, innovative strategies should be explored including glial cell reprogramming (e.g., tamoxifen derivatives inducing neuroprotective astrocytes) and microbial-mitochondrial axis interventions, combined with high-throughput screening of BBB-protective agents using organoid models. Finally, by integrating cutting-edge technologies such as fungal epigenetic regulation, engineered probiotics for immune activation, and AI-powered prognostic algorithms, a paradigm shift toward precision medicine—transitioning from pathogen eradication to neurological functional restoration—can be achieved.
